# Understanding the Associations between Smoking-Related Risk Perception, Interest in Quitting Smoking, and Interest in Lung Cancer Screening among Homeless Adult Smokers

**DOI:** 10.3390/ijerph17238817

**Published:** 2020-11-27

**Authors:** Pooja Agrawal, Matthew Taing, Tzu-An Chen, Sean M. Reuven, Michael S. Businelle, Darla E. Kendzor, Eric H. Bernicker, Lorraine R. Reitzel

**Affiliations:** 1 School of Medicine, University of Texas Medical Branch at Galveston, Galveston, TX 77555, USA; poagrawa@utmb.edu; 2 Department of Psychological Health and Learning Sciences, University of Houston, 3657 Cullen Blvd Stephen Power Farish Hall, Houston, TX 77024, USA; mtaing@central.uh.edu (M.T.); tchen3@central.uh.edu (T.-A.C.); seanreuven99@gmail.com (S.M.R.); 3 HEALTH Research Institute, University of Houston, 4849 Calhoun Rd., Houston, TX 77024, USA; 4 Health Promotion Research Center, The University of Oklahoma Health Sciences Center, 655 Research Parkway, Suite 400, Oklahoma City, OK 73104, USA; michael-businelle@ouhsc.edu (M.S.B.); darla-kendzor@ouhsc.edu (D.E.K.); 5 Houston Methodist Cancer Center, Houston, TX 77030, USA; bernicker@houstonmethodist.org

**Keywords:** lung cancer, homeless, smoking, lung cancer screening

## Abstract

Individuals experiencing homelessness smoke cigarettes at high rates, suffer a disproportionate incidence of lung cancer, but are unlikely to be screened to enhance early detection. Understanding correlates of lung cancer screening (LCS) interest within this vulnerable group may lend insight into prevention and treatment efforts and reduce their smoking-related morbidity and mortality. This study sought to understand how risk perception and interest in quitting smoking relate to LCS interest among homeless adults. Participants comprised a convenience sample of CO-verified current smokers (*N* = 310; 72.6% men, M_age_ = 43 + 11.7) from a homeless shelter in Dallas, TX. Participants self-reported risk perception, interest in quitting smoking, and interest in LCS. The average risk perception was 6.7 + 3.2 (range 0–10), 74.8% (*n* = 232) agreed or strongly agreed with interest in LCS, and 65.8% (*n* = 204) were interested in quitting smoking. Greater interest in quitting smoking, but not greater risk perception, was associated with greater interest in LCS (adjusted OR: 1.968, (95% CI: 1.213, 3.191), *p* = 0.006). Risk perception and interest in quitting smoking did not interact in their association with interest in LCS. Results suggest that homeless smokers with an interest in quitting may be receptive to LCS: a diagnostic tool by which cancers can be caught at earlier stages and prior to metastasis. However, few in the current sample would be eligible for LCS based on current guidelines; results have implications for altered screening practices among chronic smokers experiencing homelessness.

## 1. Introduction

Over 500,000 individuals experience homelessness in the United States (US) on a given night [1]. Despite having largely limited financial means [2,3], homeless adults have high rates of cigarette use, which contributes to poor health outcomes among this population [4]. Previous research has found smoking prevalence estimates for homeless individuals to be between 70–80%, a rate that is at least five times higher than in domiciled adults [4,5]. Cigarette smoking is the primary risk factor for developing lung cancer, the second most commonly diagnosed cancer among adults in the US [6], and it has significant impacts on cell cycle progression and pulmonary function [7,8]. Based on their high smoking rates, it is not surprising that one of the more comprehensive studies on morbidity and mortality among individuals who were homeless found that lung cancer caused more than one third of their deaths [9].

Given the direct causal link between smoking and the development of lung cancer, beginning in 2013, the United States Preventive Services Task Force (USPSTF) recommended annual low-dose computed tomography (LDCT) lung cancer screening (LCS) for adults who have a 30 pack-year smoking history and currently smoke or have quit within the past 15 years [10]. Although LDCT has been demonstrated to reduce lung cancer mortalities by 20%, less than 15% of eligible adults are actually screened for lung cancer annually [11,12]. Given known barriers to accessing cancer screenings and care amongst individuals experiencing homelessness, it is likely that these individuals—despite being at elevated risk for lung cancers—are not well represented among those receiving LCS [13,14]. Illustrative of this point are studies that have found high levels of advanced stage disease (78%) and metastases (68%) at diagnosis among homeless men with lung cancers [14]. Moreover, relative to domiciled adults with non-small cell lung cancer (NSCLC), adults experiencing homelessness with NSCLC had a longer span of time between abnormality identification and biopsy (248 days vs. 116 days) and a shorter survival time (0.58 years vs. 1.30 years), potentially due to disparities in receipt of timely care due to a significantly greater proportion of missed medical appointments post-diagnosis (26% of appointments missed vs. 16% of appointments missed) [15]. Consequently, better engagement of homeless individuals in early lung cancer detection efforts may be needed to improve their morbidity and mortality outcomes [14].

Little is known about correlates of interest in LCS among homeless smokers. Previous research conducted among domiciled adults has found that greater smoking-related risk perception is linked with greater interest in LCS [16,17,18,19,20]. Moreover, interest in quitting smoking has also been linked with interest in LCS among housed adults [16,17]. However, the literature is mixed, with at least one study finding null relations between LCS interest and perceived worry/risk and chance of quitting smoking [21]. Among homeless smokers, it may be that smoking-related risk perception and interest in quitting smoking each directly relate to LCS interest, or they may interact with one another to predict interest in LCS. This information might be helpful for understanding how to enhance motivation for LCS among homeless smokers, by enhancing risk perception about smoking-related diseases and/or interest in quitting smoking. Moreover, research with domiciled adults suggests that LCS may serve as a teachable moment for smoking cessation and has been linked to higher motivation to stop smoking [22,23,24]. Thus, better understanding the correlates of LCS interest among homeless smokers may help to enhance both prevention and screening efforts in this group. Consequently, the purpose of the current study was to explore links between smoking-related risk perception, interest in quitting smoking, and interest in LCS among a sample of homeless adult smokers.

## 2. Materials and Methods

### 2.1. Participants and Procedures

Study procedures were approved by the Institutional Review Board (IRB) at the University of Texas Health Science Center at Houston (HSC-SPH-13-0277), approval date 5/22/13, and the University of Houston (13577-EX), approval date 8/30/13. Participants (*N* = 394) in the parent study comprised a convenience sample of homeless adults who were recruited from a large shelter in Dallas, TX, USA in 2013. This shelter was selected as a recruitment site based on its service to 85% of individuals experiencing homelessness in Dallas and due to a long-standing collaboration with the co-authors of this study [25,26]. Participants were recruited through two waves of data collection for a study that was primarily designed to assess the impact of a partial smoking ban at the shelter but that also assessed health and health behaviors [27]. The partial smoking ban entailed making half of the courtyard within the confines of the shelter’s grounds a smoke-free zone. Data were collected prior to the partial ban (wave 1; June 2013) and following the partial ban (wave 2; August 2013). Recruitment was accomplished via flyer advertisement. Inclusion criteria were: aged 18 or over, English-speaking and literate at the 7th grade level or more as indicated by a score of >4 on the Rapid Estimate of Adult Literacy in Medicine—Short Form [28], and having spent at least the prior night at the shelter. Screening, written informed consent, and data collection were conducted on site. Participants received a USD 20 gift card as remuneration for their participation in either or each wave of data collection completed.

### 2.2. Measures

#### 2.2.1. Participant Characteristics

Participant sociodemographic variables included age, sex, race (white versus minority race), education (in years; also quantified as GED/high school diploma or less versus some college/technical school or more), last month’s income (USD 0, 1–500, >500), employment status (at least part-time employed versus unemployed), health insurance status (any type versus none), and lifetime homelessness (in months).

Participant smoking-related variables included smoking rate (“How many cigarettes a day do you smoke on average?”), number of years smoked over the lifetime, and how soon after waking is the first cigarette smoked (within 5 min, 5–30 min, 31–60 min, or >60 min).

#### 2.2.2. Smoking-Related Risk Perception

Smoking-related risk perception was measured with the question: “What are the chances of developing at least one smoking related disease if you do not quit smoking?”. This item was measured from 0%, which had the anchor “I definitely will not develop” to 100%, which had the anchor “I definitely will develop”. The option of 50% had the anchor of “I have a 50/50 chance”. Participants could select any number between 0% and 100% in increments of 10, and their responses were coded from 0–10 in the database.

#### 2.2.3. Interest in Quitting Smoking

Interest in quitting smoking was assessed using a single item reading: “I would like to stop smoking”. Answer options were 0 = no or 1 = yes.

#### 2.2.4. Interest in Lung Cancer Screening

Interest in LCS was an investigator generated single item reading: “I would be interested in taking a test that can screen for lung cancer”. Answer options were scored 1 to 5, where 1 = strongly disagree, 2 = disagree, 3 = neither agree nor disagree, 4 = agree, and 5 = strongly agree. The first two categories were collapsed into a single “disagree” category due to low frequencies of these two responses (*n* = 26 and *n* = 20, respectively).

### 2.3. Statistical Analysis

The current analyses are limited to the current smokers in the sample (310/394 = 78.7%), defined as having smoked at least 100 cigarettes over the lifetime and endorsing current smoking every day or some days during the week [29]. The restriction to this dataset was necessary, as some items of interest (e.g., interest in quitting smoking) were only administered to those who were current smokers. Data were explored using descriptive statistics. Sample comparisons based on interest in quitting smoking were performed using independent t-tests or chi-square tests for continuous and categorical variables, respectively. Ordinal logistic regression models were used to estimate associations of smoking-related risk perception, interest in quitting smoking, and interest in LCS, controlling for wave of data collection, smoking rate, age, sex, race, and education (in years). Additionally, the moderation effect of interest in quitting smoking was examined via an interaction term following mean-centering of variables. The chi-square score test for the proportional odds assumption was employed to examine whether the ordinal regression model assumption was violated or not. All analyses were conducted using SAS version 9.4 in June 2020 [30]. Alpha was set at 0.05.

## 3. Results

The average risk perception was 6.7 + 3.2 (range 0–10), 74.8% (*n* = 232) agreed or strongly agreed with interest in LCS, and 65.8% (*n* = 204) were interested in quitting smoking (see Table 1). Significant differences between those who were interested in quitting or not were found in age (*p* = 0.03), race (*p* = 0.01), employment status (*p* = 0.047), and interest in LCS (*p* = 0.042). Participants who were interested in quitting were older (44.28 vs. 41.03), more likely to be of a minority race (74.51% vs. 60.38%), unemployed (92.16% vs. 84.91%), and more likely to be strongly interested in LCS (30.88% vs. 16.98%, *p* = 0.0082). The proportional odds assumption was satisfied (*p* > 0.05).

As seen in Table 2, greater risk perception was not significantly associated with interest in LCS (adjusted odds ratio (OR): 1.043, (95% CI: 0.927, 1.174), *p* = 0.48); however, interest in quitting smoking was significantly associated with interest in LCS (adjusted OR: 1.968, (95% CI: 1.213, 3.191), *p* = 0.006). The expected odds of greater interest in LCS were significantly greater for those who were interested in quitting smoking. However, the association of risk perception and interest in LCS was not moderated by interest in quitting smoking (*p* = 0.418).

## 4. Discussion

Among this sample of adult homeless smokers, interest in quitting smoking was significantly associated with interest in LCS, a finding that has been reported in previous studies among domiciled adults [16,17]. The current study extended these results to a sample of homeless adults, a group with high rates of smoking and smoking-related disease, including lung cancers [2,4,9,31,32,33]. Results suggest that homeless smokers with an interest in quitting may be receptive to LCS: a diagnostic tool by which cancers can be caught at earlier stages and prior to metastasis [11,12]. This is important given that even routine cancer screenings are not undertaken as recommended among this group [13], and in particular because their lung cancers are typically caught at later stages of disease relative to their domiciled counterparts [14]. Thus, chronic homeless smokers with an interest in quitting smoking might also be screened for LCS eligibility and provided with a practical means by which to obtain it so as to enhance early detection. Moreover, interest in quitting smoking and interest in cancer screenings are each potentially malleable; therefore, future work should examine if interest in both can be further enhanced via, for example, brief motivational interviewing interventions with LCS-eligible homeless smokers [34,35,36]. Finally, there may be a reciprocal relationship between interest in quitting smoking and interest in LCS whereby LCS serves as a “teachable moment” that allows providers to initiate conversations with patients to reinforce their desire to quit [9,10,11]. Previous studies have found that after receiving LCS, the quit rate ranged from 11.9% to 15.5% [37], a rate much higher than the percentage of US adult smokers who successfully quit smoking in the past year (7.5%) [38]. As such, increasing access to LCS among eligible homeless smokers may further increase motivation to quit smoking and thereby help engender successful quit attempts. The current study was cross-sectional, however, and future longitudinal work is needed to delineate causal and potentially reciprocal pathways between interest in quitting smoking, interest in LCS, and LCS impacts on smoking cessation among this vulnerable group.

Contrary to some previous findings [16,17,18,19,20], risk perception was not significantly associated with interest in LCS. These results, however, may be expected given that many homeless people experience significant barriers to cancer screening that potentially detract from the perceived importance of early detection and prevention [39]. Factors such as low socioeconomic status may bar homeless patients from seeking LCS due to an inability to pay for a screening and follow-up care [39]. In a previous study conducted among domiciled adults, intention to screen for lung cancer dropped by 50% when participants were told that the screening was only available at their own expense [40]. One recent study has found that out-of-pocket LCS costs for uninsured patients were highly variable (range USD 49 to 2409) [41]. This variability may influence LCS participation rates among uninsured patients. Further, barriers related to housing status and food insecurity may detract from screening interest [13,42]. The importance of early detection and the role of LCS may also not be sufficiently understood among this population, which has low rates of health literacy and limited education compared to the general population [43,44]. In addition to a lack of access to a usual source of primary care [45], homeless individuals may receive infrequent tobacco use education during the limited healthcare they receive [46], further suggesting that homeless smokers may not be fully aware of the risks related to smoking and the relationship between smoking and disease incidence and thus would not be able to make informed perceptions of risk or the need to quit. Healthcare providers also often adopt fatalistic attitudes towards this population that consequentially normalize and reinforce smoking and detract from cessation efforts among homeless smokers [47]. These structural barriers to preventative care [9], coupled with competing priorities of day-to-day survival needs [48], may then mitigate LCS salience and interest among individuals experiencing homelessness. This paucity of screening coupled with disproportionate cancer mortality rates and increased late stage cancer diagnoses [9] indicate a need for system-wide interventions to increase access to preventative care, including LCS. Future studies should thus employ a qualitative approach to further delineate homeless smokers’ tobacco-related risk perceptions, beliefs regarding LCS benefits/barriers, and barriers to smoking cessation. Addressing existing misconceptions and barriers may ultimately encourage positive health behaviors such as smoking cessation and interest in pursuing LCS [49,50,51,52,53,54].

At the time of data collection, available guidelines for LCS consisted only of those from the American Association for Thoracic Surgery [55]; guidelines from the USPSTF [56] were released shortly after our data collection. Notably, in both cases, these guidelines (e.g., age 55–80 with ≥30 pack-year smoking history) exclude many of our study’s participants. In total, only 10 persons in our sample (3.23%) were eligible for LCS screening, at least based on their current smoking rate. However, the USPSTF is currently considering an update to the guidelines that would reduce age requirements from 55–80 to 50–80 and reduce the pack-year requirement from 30 to 20. If the new guidelines are approved, 32 participants (10.32%) would meet the eligibility criteria for screening. It should be noted that interest in LCS and eligibility for LCS are discrete concepts and homeless individuals are an extremely destitute group, with high rates of unemployment and low rates of insurance coverage [2,3]. Therefore, assessing pack-years using average cigarettes per day may reflect monetary availability and may very well underestimate chronic exposure to combustible tobacco accrued over time and prior to homelessness. Moreover, nicotine addiction may be satisfied by various tobacco products while experiencing homelessness, rendering cigarettes smoked per day in an average week not representative of prior or desired consumption. To this point, 49.49% of this study’s participants indicated that they now smoke fewer cigarettes per day than they did a year ago. Likewise, other research suggests high rates (e.g., 67.2%) of concurrent tobacco product use amongst smokers experiencing homelessness [31,33]. Additionally, as a result of limited financial resources, homeless smokers frequently adopt alternative smoking behaviors, such as borrowing cigarettes, sharing cigarettes, and smoking discarded cigarette butts/filters (in addition to traditional methods of smoking) [2,3,52]. These opportunistic smoking behaviors may then be inaccurately represented in traditional measures for cigarettes smoked per day and consequentially in a pack-year. Furthermore, although homeless adults have higher rates of current tobacco use than domiciled populations, one study has found that there was no difference in total pack-years between homeless and housed groups [15,57]. Given that homeless smokers experience high rates of cancers of the lung [9], current criteria for LCS—or at least the way smoking history is assessed and considered—may need to be altered for individuals experiencing chronic homelessness, given that reported smoking history or pack-years may not accurately reflect exposure [15]. As such, it may be more valuable to implement individualized risk-based selection that considers personal demographic, clinical, and smoking characteristics, as opposed to using standardized guidelines, to determine screening eligibility [58,59,60]. Personalized risk-based selection has been demonstrated to be more effective in preventing lung cancer deaths than selecting for subgroups per current USPSTF recommendations [61,62]. Thus, implementing personalized risk-based screening strategies can potentially capture USPSTF-ineligible high-risk groups in addition to those who are currently eligible. Revising current guidelines to be more inclusive of homeless smokers and other non-traditional smokers cannot be effective without proper access to LCS and smoking cessation products. With regard to the former, although costs and lack of insurance are commonly endorsed barriers to screening, the Centers for Medicare and Medicaid services has recently approved coverage for LDCT scans among eligible adults, which may broaden its availability to low income groups [11]. With regard to the latter, some homeless serving agencies are beginning to embrace the provision of evidence-based smoking cessation services to their guests/clients [63]. Taken together, increased systematic support for healthcare and revised screening guidelines could potentially help to mitigate existing disparities among vulnerable groups such as homeless smokers.

Study limitations include those relevant to generalizability of results given the use of a convenience sample from a single shelter in Dallas, TX. Although this sample is relatively small (*N* = 310) and consists of more men (72.58%) than are represented in the overall US homeless population, it represents a demographic that is both sex-matched and racially similar to Dallas’ overall homeless adult population [64]. Additionally, although evidence-based smoking cessation interventions are not commonly provided to individuals experiencing homelessness, the shelter from which the participants were recruited did offer such services [4,26,47]. Thus, results may not be generalizable to individuals sheltering in centers without these resources. Moreover, we did not have access to data regarding who from our sample might be participating in the shelter’s smoking cessation services to adjust or otherwise account for this factor. Other limitations include the use of self-reported measures of risk perception and interest in LCS, which may be affected by bias. Furthermore, participants were only asked about interest in LCS and were not given information about related costs or procedures associated with LCS. In some ways, this may have avoided the influence of contextual factors such as access and expense on responses; however, responses cannot be considered a proxy for future intentions to participate in LCS or continued interest in LCS once potential access and financial challenges were explicated. Our risk perception assessment for smoking-related disease was limited to current smokers; thus, former smokers who might be eligible for LCS were not included in our analyses. Furthermore, risk perception related to other areas (e.g., safety, financial struggle, access to food, treatments for existing disease) was not assessed and therefore could not be examined in relation to risk perception for a smoking-related disease. The single measure of risk perception may benefit from additional measures that capture more detailed assessments of tobacco-related risks (i.e., cancer, respiratory diseases). Additionally, although we controlled for some variables in our analysis, additional factors not accounted for or assessed in this study (e.g., certain chronic conditions, experience with prior cancer screenings, smoking-related disease knowledge) may have affected the primary variables of interest or their association in unknown ways. Replication is needed. Lastly, the cross-sectional nature of this study excluded any assumptions regarding bidirectionality or causality. Future studies should seek to extend exploration for bidirectionality and causality within the contexts of smoking-related risk perception, interest in quitting smoking, and interest in LCS.

## 5. Conclusions

In summary, this study expands upon existing literature by supporting the link between interest in quitting and interest in LCS among a sample of homeless adults. These findings suggest that homeless smokers with an interest in quitting may be receptive to LCS. This is important because LCS may reduce lung cancer disparities within this group via early detection [9,14]. However, few smokers in the current sample would be eligible for LCS based on current guidelines; thus, results have implications for altered/personalized screening practices among chronic smokers experiencing homelessness. Implementing changes to existing guidelines to account for individual risk-based factors, coupled with increasing access for LCS and to evidence-based tobacco control and smoking cessation products, could help facilitate early detection and treatment of lung cancer among high risk, largely USPSTF-ineligible groups such as smokers experiencing homelessness.

## Figures and Tables

**Table 1:** Participants’ characteristics by interest in quitting smoking.

Sociodemographic Variables	Interest in Quitting Smoking			
	Total	No	Yes	*p*-Value
	310	106 (34.19%)	204 (65.81%)	
	N (%)/M [SD] ^a^			
Age	43.17 [11.69]	41.03 [13.03]	44.28 [10.80]	0.0287
Sex				0.1111
Female	85 (27.42)	35 (33.02)	50 (24.51)	
Male	225 (72.58)	71 (66.98)	154 (75.49)	
Race				0.0102
White	94 (30.32)	42 (39.62)	52 (25.49)	
Minority	216 (69.68)	64 (60.38)	152 (74.51)	
Last month’s income				0.6978
$0	128 (44.14)	49 (47.12)	79 (42.47)	
$1–$500	88 (30.34)	31 (29.81)	57 (30.65)	
>$500	74 (25.52)	24 (23.08)	50 (26.88)	
Employment status				0.0465
At least part-time employed	32 (10.32)	16 (15.09)	16 (7.84)	
Unemployed	278 (89.68)	90 (84.91)	188 (92.16)	
Education (in years)	11.82 [1.58]	11.88 [1.57]	11.79 [1.58]	0.6385
Education level				0.2908
GED/high school diploma or less	225 (72.58)	73 (68.87)	152 (74.51)	
Some college/technical school or more	85 (27.42)	33 (31.13)	52 (25.49)	
Health insurance				0.3111
Any type health insurance	78 (25.16)	23 (21.70)	55 (26.96)	
No health insurance	232 (74.84)	83 (78.3)	149 (73.04)	
Lifetime homelessness (in months)	39.34 [50.23]	36.81 [35.59]	40.69 [56.57]	0.4648
Data collection wave				0.9392
Wave 1	191 (61.61)	65 (61.32)	126 (61.76)	
Wave 2	119 (38.39)	41 (38.68)	78 (38.24)	
**Smoking-Related Variables**	**N (%)/M [SD] ^a^**			
Smoking rate (avg. cigarettes per day)	12.02 [7.17]	12.98 [6.90]	11.56 [7.27]	0.1144
Years of smoking over the lifetime	19.14 [11.90]	19.75 [12.36]	18.84 [11.69]	0.5436
Average pack-years ^╪^	12.03 [11.50]	13.49 [12.23]	11.35 [11.11]	0.1386
How soon after waking do you smoke your first cigarette?				0.7638
Within 5 min	104 (35.49)	35 (36.46)	69 (35.03)	
5–30 min	98 (33.45)	35 (36.46)	63 (31.98)	
31–60 min	39 (13.31)	11 (11.46)	28 (14.21)	
After 60 min	52 (17.75)	15 (15.63)	37 (18.78)	
Smoking-related risk perception (range: 0–10)	6.70 [3.17]	6.22 [3.22]	6.93 [3.13]	0.072
Interest in lung cancer screening				0.0416
Strongly disagree	26 (8.39)	10 (9.43)	16 (7.84)	
Disagree	20 (6.45)	10 (9.43)	10 (7.90)	
Neither agree or disagree	32 (10.32)	15 (14.15)	17 (8.33)	
Agree	151 (48.71)	53 (50.00)	98 (48.04)	
Strongly agree	81 (26.13)	18 (16.98)	63 (30.88)	

^a^ SD: standard deviation. ^╪^ pack-years = # of cigarettes smoked per day/20 × number of years smoked.

**Table 2:** Results of interest in lung cancer screening from ordinal regression analysis.

Variables in Analysis	Odds Ratio	95% CI ^a^	*p*-Value
Age	0.981	(0.962, 1.000)	0.051
Sex (Ref: female)	1.448	(0.884, 2.373)	0.142
Race (Ref: minority)	1.256	(0.757, 2.085)	0.377
Education (in years)	1.166	(1.012, 1.343)	0.033
Data collection wave (Ref: wave 1)	1.117	(0.698, 1.786)	0.645
Smoking rate (average cigarettes smoked per day)	0.980	(0.949, 1.012)	0.211
Smoking-related risk perception	1.043	(0.927, 1.174)	0.480
Interest in quitting smoking (Ref: no)	1.968	(1.213, 3.191)	0.006
Smoking-related risk perception * Interest in quitting smoking	1.062	(0.918, 1.228)	0.418

^a^ CI: confidence interval. * = moderation term.
